# Insight into Pyrolysis Behavior and Cross-Linking Reactions Mechanism During Coking Coals Pyrolysis

**DOI:** 10.3390/ma19061096

**Published:** 2026-03-12

**Authors:** Lu Tian, Jinxiao Dou, Xingxing Chen, Jianglong Yu

**Affiliations:** 1 Key Laboratory of Advanced Coal and Coking Technology of Liaoning Province, School of Chemical Engineering, University of Science and Technology Liaoning, Anshan 114051, China; 320163300148@ustl.edu.cn (L.T.); xingchenstar79@163.com (X.C.); 2 Suzhou Industrial Park Monash Research Institute of Science and Technology, Southeast University-Monash University Joint Graduate School, Suzhou 215123, China

**Keywords:** coking coal, pyrolysis, cross-linking reaction, gas component, tar composition

## Abstract

**Highlights:**

- The remains of aliphatic structures within the plastic layer promotes cross-linking reactions during the softening–melting stage.
- (C–C)/(C–H) ratio and H_2_ evolution is a key indicator of aromatization cross-linking intensity.
- Structural densification and aromatic growth after resolidification confirm the intensification of cross-linking, driving the formation of coke.

**Abstract:**

Coke, as an essential metallurgical raw material, is widely used in iron and steel production. To investigate the pyrolysis behavior and cross-linking reactions during the pyrolysis of coking coal, pyrolysis experiments were conducted in a quartz-tube fixed-bed reactor placed in an electric furnace. The yields and compositions of the pyrolysis products were systematically analyzed. Gaseous and tar components generated at different pyrolysis stages were characterized using gas chromatography (GC) and gas chromatography–mass spectrometry (GC–MS). The semi-coke was examined by X-ray photoelectron spectroscopy (XPS) and scanning electron microscopy (SEM). The results indicated that the yields of tar from coking coal pyrolysis have a notable impact on the cross-linking reactions occurring during the coal pyrolysis process. The structural differences between Malan coal (ML) and Tunlan coal (TL) coals underlie their distinct behaviors in cross-linking intensity, tar evolution profiles, and coke-forming properties. For high-volatile, highly fluid ML coal, the release of the aliphatic compounds in tar volatiles remains relatively low at the temperature of maximum fluidity, which is beneficial to the cross-linking reactions. In contrast, for TL coal with lower volatility and fluidity, substantial H_2_ emission during the early pyrolysis stage promotes cross-linking reactions. This study provides new insights into the temperature-dependent evolution of cross-linking reactions during coking coal pyrolysis.

## 1. Introduction

Coking coal serves as an essential raw material in the metallurgical industry, with its pyrolysis behavior critically influencing plasticity development, structural transformation, and the ultimate properties of coke [1,2]. During pyrolysis, coking coal undergoes a series of complex physicochemical processes, including functional group decomposition, aliphatic side-chain scission [3,4], radical generation [5], cross-linking reactions, and aromatic condensation [6,7,8]. Among these processes, cross-linking reactions play a pivotal role in bridging molecular-scale structural evolution with mesoscale carbon organization and macroscopic coke performance. Early mechanistic studies have proposed that cross-linking reactions arise from radical recombination and dehydrogenative condensation during pyrolysis, leading to the formation of increasingly condensed aromatic networks [6]. Nevertheless, the formation mechanism, evolutionary pathway, and structural implications of cross-linking reactions during coking coal pyrolysis have not yet been fully elucidated.

Recent studies have systematically investigated the structural evolution of carbon in coking coal during pyrolysis across a range of temperatures. Hu et al. [9,10] utilized a customized TG-P-SP apparatus to characterize the plastic layer and semi-coke layer formed during the coking process, which helped clarify their correlation with coal thermoplasticity and coking properties. Analytical techniques such as SEM, Fourier Transform Infrared Spectroscopy (FTIR), Raman Spectroscopy, and X-ray Diffraction (XRD) have been employed to examine both physical and chemical transformation mechanisms, including cleavage of aliphatic bonds and condensation of aromatic rings. Wang et al. [11] employed a multi-analytical approach (XRD, Raman, and FTIR) to demonstrate that the medium- to high-rank coals exhibit increased aromaticity, reduced interlayer spacing of aromatic structures, and a gradual loss of oxygen-containing functional groups.

The chemical nature of these bonds during the transition phase is critical. Chen et al. [12] identified that above the resolidification temperature, the transformation of C–O and C–H bonds into C–C bonds was accompanied by the release of H_2_ and CO_2_, promoting greater structural ordering and anisotropy in metallurgical coke. Furthermore, Xu et al. [7] systematically elucidated the development of both carbon and cross-linking structures, demonstrating through High-Resolution Transmission Electron Microscopy (HRTEM) and XPS that the progressive formation of cross-linked aromatic networks plays a critical role in carbon ordering and in determining final coke quality.

Beyond the static coal matrix, the role of mobile phases and volatiles in the pyrolysis mechanism cannot be overlooked. Cao et al. [13] demonstrated that thermally soluble fractions initially act as lubricants to promote coal softening and enhance coal fluidity. However, at higher temperatures, these fractions became reactive sites for secondary condensation, contributing to the rigidity of the carbon framework. Pyrolysis volatiles are an essential factor affecting the pyrolysis process and the mechanism of coal. Cui et al. [14] found that aromatic clusters with 3–5 rings are the primary components that promote the thermoplastic properties and development of fluidity for coking coals, while Li et al. [15] observed that the secondary reactions of volatile radical fragments with char surfaces lead to increased coke yield at the expense of tar quality. More recently, Zhao et al. [16] studied the free radical concentration and gas release influence coal’s coking behavior and cross-linking structure. Results showed that in the formation of the plastic layer, the cleavage of fatty side chains and oxygen-containing functional groups produces CH_4_ and CO_2_. After that, the aromatic clusters begin polycondensation reaction and produce a large amount of H_2_ [12,16]. The gas volatiles were paid attention to, and the tar composition was lower.

In this study, pyrolysis experiments of coking coals were conducted at a temperature range of 400–900 °C. By systematically analyzing the composition of the tar, gas, and the structure of semi-coke at different temperatures, the mechanisms of coal pyrolysis were investigated. Understanding the pyrolysis behavior and cross-linking reactions of coking coal provides insight into the fundamental coking mechanism and its transformation during the coking process. This knowledge provides a theoretical basis for coke quality control and the optimization of coking processes to improve industrial performance.

## 2. Experimental Section

### 2.1. Coal Samples Preparation and Analysis

Two Chinese coking coals were used in this study. The coking coals were sieved to obtain particles sized less than 1.5 mm. The proximate analysis, ultimate analysis, and coal properties were shown in Table 1, according to the test standards (GB/T 212-2008 [17], GB/T 25213-2010 [18], GB/T 8899-2013 [19]). Coal samples had different volatiles, maceral composition, and fluidity. ML is a mid-volatile coal, with a higher vitrinite content (60.2%) and a higher maximum Gieseler fluidity (α_max_) of 2500 dd/min. TL has the highest rank, with a vitrinite content of 48%, exhibiting a maximum Gieseler fluidity of 500 dd/min. ML has the higher oxygen content (4.56 wt%, daf) in this study.

### 2.2. TG-DTG Analysis of Samples

The coal samples were measured by a STA 449 F3 thermogravimetric analyzer (TG, Netzsch, Selb, Germany). During each test, about 40 mg of coal sample was used, and it was heated in an alumina ceramic crucible under a continuous argon flow of 60 mL/min from 30 °C to 1000 °C with the heating rate of 10 °C/min. The Thermogravimetric Analysis (TG), derivative thermogravimetry (DTG) curves, and the volatiles were analyzed to characterize the thermal properties of the coal.

### 2.3. Experimental Setup and Methods

#### 2.3.1. Pyrolysis Experimental Setup and Methods

**Figure 1:**
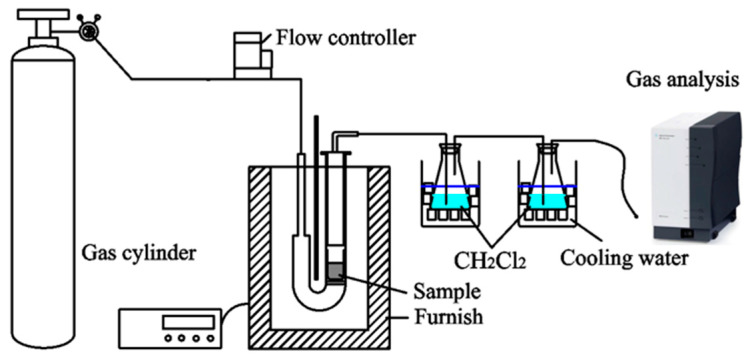
A schematic of the pyrolysis experimental setup.

Coal pyrolysis was performed on the apparatus shown in Figure 1, which includes a 15 mm diameter quartz tube reactor. In each experiment, 5 g of the coal sample was placed in the quartz reactor in an electrical furnace. The sample was heated at a rate of 3 °C/min to the target temperatures (T_i_, T_m_, T_r_, 700 °C, and 900 °C) with a holding time of 5 min. Nitrogen was used as the carrier gas at a flow rate of 100 mL/min. Coal samples were pyrolyzed at each temperature to analyze the product yields and compositions during the pyrolysis process. Two gas-washing bottles loaded with dichloromethane (CH_2_Cl_2_, Sinopharm Chemical Reagent Co., Ltd., Shanghai, China) were placed in an ice bath to collect condensable volatiles. During pyrolysis, the condensable tar was trapped in the dichloromethane, while non-condensable gases were collected using an aluminum foil gas bag. The light gases were analyzed promptly by gas chromatography. After cooling, the solid char residue was collected and weighed. The liquid tar accumulated in the gas-washing bottles, transfer lines, and reactor was dissolved in dichloromethane and subsequently filtered to eliminate any entrained char particles. Tar was subsequently separated from dichloromethane by distillation at 40 °C to determine its yield. Dichloromethane exhibits high solubility for tar, which simplifies handling and enhances the accuracy of subsequent identification of tar components by GC–MS analysis. Each experiment was performed in triplicate.

#### 2.3.2. The Yield of Pyrolysis Products

The mass of char and tar was weighed. The mass of the gas product was determined by subtracting the combined weights of the char and tar from the initial sample weight. The yield of pyrolysis products was calculated by Equations (1)–(3), where m_char_, m_tar_, and m_gas_ represent the weight of char, tar, and gas. Yield_char_, Yield_tar_, and Yield_gas_ represent the yield of char, tar, and gas, respectively [5]. The yield of volatiles is the sum of the yields of tar and gas.

(1) $${Yield}_{char}=\frac{m_{char}}{m_{coal}}\times 100\%$$

(2) $${Yield}_{tar}=\frac{m_{tar}}{m_{coal}}\times 100\%$$

(3) $${Yield}_{gas}=\frac{m_{coal}-m_{char}-m_{tar}}{m_{coal}}\times 100\%$$

#### 2.3.3. Tar Products Analysis for Pyrolysis

The tar product collected from the wash bottles was distilled to remove the solvent. The composition of tar was analyzed using a gas chromatograph and mass spectrometer (GC–MS, ISQ, Thermo Fisher Scientific Inc., Waltham, MA, USA) equipped with a capillary column with a length of 30 m and a diameter of 0.25 mm, and an electron impact (70 eV) mode. The temperature of the GC oven was ramped from 40 to 100 °C at 10 °C/min, at a heating rate of 15 °C/min from 100 to 280 °C, and with a holding time of 15 min. Helium was used as the carrier gas during the GC–MS measurements. The split ratio was 20:1, and the inlet temperature of the column was 280 °C. A mass range of 50–500 amu for mass spectrometry was applied. The chemical compounds in the tar were identified by comparison with the NIST11 mass spectral library database in the instrument database. A semi-quantitative analysis was compared with the composition of pyrolysis tar. For semi-quantitative analysis, the area normalization method was employed. The relative content (area %) of each compound was calculated as the ratio of the peak area of the individual compound to the total peak area of all detected compounds. Peaks with a signal-to-noise ratio (S/N) greater than 3 were included in the analysis. Therefore, the reported area percentages represent relative abundances rather than absolute mass fractions, and the results are used mainly for comparative analysis of tar composition between different coal samples and pyrolysis conditions.

#### 2.3.4. Gas Products Analysis for Pyrolysis

The light gaseous species including hydrogen (H_2_), methane (CH_4_), carbon monoxide (CO), carbon dioxide (CO_2_), and C_2_–C_3_ hydrocarbons (ethane (C_2_H_6_), ethylene (C_2_H_4_), and propane (C_3_H_8_)) were analyzed using a gas chromatograph (Micro-GC 490, Agilent, Santa Clara, CA, USA) equipped with a thermal conductivity detector. The system featured two independent analytical channels, one containing an MS5A (Molecular Sieve 5A) column and the other a PPU (Polar Plot U) column. High-purity argon (99.999%) was employed as the carrier gas for the MS5A column to separate H_2_, CH_4_, and CO, while high-purity helium (99.999%) was used as the carrier gas for the PPU column to analyze CO_2_ and C_2_–C_3_ hydrocarbons. The MS5A and PPU columns were operated at 85 °C and 120 °C, respectively, with inlet pressures maintained at 150 kPa and 120 kPa. The injector temperature was set to 80 °C. Prior to analysis, the instrument was calibrated using certified standard gas mixtures containing known concentrations of H_2_, CH_4_, CO, CO_2_, C_2_H_6_, C_2_H_4_, and C_3_H_8_. Each analytical run required a total duration of 2 min to measure the composition of the gaseous products.

#### 2.3.5. Char Products Analysis for Pyrolysis

The changes in the chemical structure of char were studied by XPS and SEM.

(1) XPS Analysis Method

The samples were analyzed by XPS using an AXIS–SUPRA instrument (Kratos AXIS SUPRA, SHIMADZU, Tokyo, Japan) in this paper, and the X-ray excitation source used was Al Kα radiation (hv = 1486.6 eV) with an X-ray beam spot of 110 μm. The samples of raw coal and semi-coke products at 400–900 °C were tested for XPS. The XPS tests were performed at a voltage of 15 kV and a current of 10 mA. Survey spectra were recorded with a pass energy of 160 eV and a step size of 1 eV, while high-resolution spectra were collected with a pass energy of 20 eV and a step size of 0.1 eV. The binding energy of C1s for XPS analysis used an electron binding energy range from 280 to 294 eV.

The C1s spectra were deconvoluted through peak fitting analysis, yielding six principal components [7,16]. Table S1 shows the assignment and electron binding energy of peaks at C1s binding energy [7,16,20,21]. The peak observed at approximately 284.6 eV corresponds to aromatic or graphitized carbon structures, specifically C–C bonds with sp^2^ hybridization. At around 285 eV, the signal was assigned to C–H bonds in substituted alkanes attached to aromatic frameworks. Peaks near 286.3 eV were attributed to C–O bonds present in phenols, alcohols, and ether groups (C–O–C). The contribution at about 287.6 eV arises from carbonyl functionalities (C=O), while the peak near 289.1 eV indicates the presence of carboxyl groups (COO–). Finally, the feature near 291 eV was associated with π–π* transitions.

(2) SEM Analysis Method

Scanning electron microscopy (SEM, ZEISS Sigma HD, Carl Zeiss AG, Oberkochen, Germany) was used to study the surface images of the samples. An accelerating voltage was 20 kV. The microscope is equipped with various detectors, such as the Secondary Electron Detector (SE) for surface morphology and the Backscattered Electron Detector (BSE) for compositional contrast.

## 3. Results and Analysis

### 3.1. TG-DTG Analysis of Coals

Pyrolysis experiments were carried out on ML and TL coals at different temperatures. Figure S1a,b illustrate the TG and DTG curves for ML and TL coals, respectively. Based on the TG–DTG profiles, the pyrolysis and decomposition behavior of the coking coals can be categorized into three distinct stages [22]. The first stage, occurring below 300 °C, corresponds to the release of moisture. Since the moisture content in these coals was less than 1%, this stage was not prominent. The primary decomposition took place between 400 and 500 °C, representing the devolatilization stage, during which the macromolecular structures were broken down, leading to the release of volatile compounds. The third stage, characterized by a slight weight loss above 700 °C, was attributed to the aromatization of semi-coke, accompanied by polycondensation and secondary reactions.

**Figure 2:**
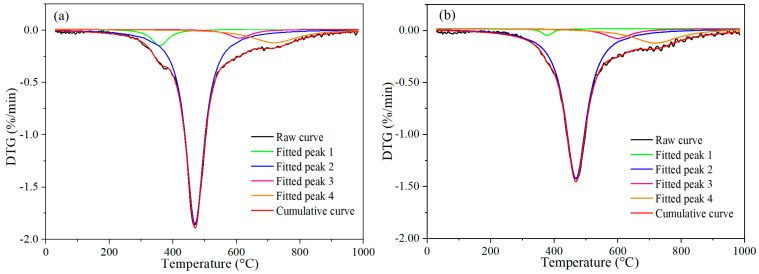
DTG results of the samples during pyrolysis: (**a**) ML; (**b**) TL.

The DTG curves were fitted to identify characteristic peaks, enabling an assessment of volatile release as a function of temperature associated with covalent bond dissociation, as shown in Figure 2. Below 300 °C, the bound water disintegrates, and the moisture of these coking coals is less. A slight weight loss occurs at 300–400 °C (Peak 1), attributed to the cleavage of C_al_–O/N/S and S–S bonds, which possess relatively low dissociation energies [21,23,24]. The principal decomposition stage takes place from 400 to 500 °C due to devolatilization. Within this range, intensive decomposition (Peak 2) corresponds to the cleavage of C_al_–C_al_, C_al_–H, C_ar_–H, C_al_–O, and C_ar_–N bonds [21,22]. A further dissociation stage appears between 500 and 700 °C (Peak 3), which can be ascribed to the fracture of C_ar_–C_al_ and C_ar_–O/S bonds [21]. Finally, in the temperature interval of 700–900 °C (Peak 4), polymerization reactions of aromatic rings occur.

### 3.2. The Distribution of Products Yields During Pyrolysis

**Figure 3:**
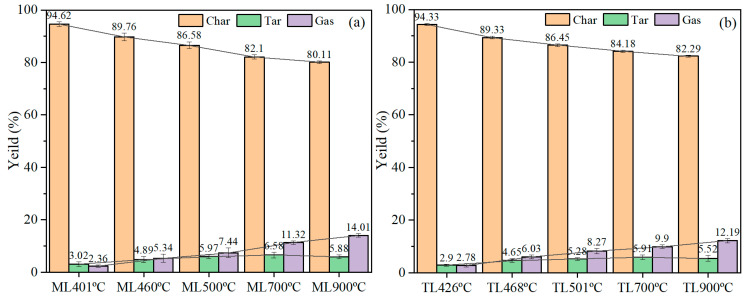
Product yields of different coking coals under different temperatures during pyrolysis: (**a**) ML; (**b**) TL.

When the samples were heated, volatiles were released, combining gaseous and tar components from coal particles; their yields were quantified under different conditions. The yields of pyrolysis products are shown in Figure 3.

Figure 3a,b show the product distributions obtained from the pyrolysis of ML and TL coals at varying temperatures over three times. The results indicate that tar yield initially increased with temperature up to 700 °C, but declined beyond 900 °C. In contrast, gas yield exhibited a continuous upward trend throughout the temperature range, while char yield showed a progressive decline. Consequently, the total volatile matter yield increased with rising pyrolysis temperature. As shown in Figure 3a, during the initial decomposition to solidification stage (400–500 °C), the tar yield of sample ML rose from 3.02% to 5.97%. Increasing the temperature from 500 to 700 °C further raised the tar yield from 5.97% to 6.58%. However, when the temperature was increased to 900 °C, the tar yield decreased to 5.88%. This decline can be attributed to secondary pyrolysis reactions, particularly the cracking of tar into light gases. Meanwhile, gas yield increased steadily with temperature, from 7.44% at 500 °C to 14.01% at 900 °C, accompanied by a monotonic decrease in char yield from 94.62% at 401 °C to 80.11% at 900 °C. The pronounced increase in gas yield above 700 °C is likely due to enhanced aromatization and cross-linking reactions that release hydrogen, as well as the secondary cracking of tar at elevated temperatures.

The product distribution from the pyrolysis of TL coal was shown in Figure 3b, with an overall trend similar to that observed for ML coal. At 501 °C, the tar yield from TL coal pyrolysis was 5.28%, increasing slightly to 5.91% at 700 °C. As the temperature further increased from 700 °C to 900 °C, the tar yield gradually decreased to 5.52%. Correspondingly, the deviation in tar yield initially increased with temperature before declining at higher temperatures. In contrast, the gas yield deviation rose steadily from 8.27% at 501 °C to 9.90% at 700 °C, and further to 12.19% at 900 °C. Above 700 °C, both tar and gas yields of TL coal were lower than those of ML coal, a finding consistent with the results of proximate and TG analyses. Conversely, the char yield exhibited an inverse trend, decreasing as the temperature increased. Below 500 °C, the tar and gas yields of the low-rank ML coal were generally higher than those of TL coal. The variation in cross-linking with pyrolysis temperature suggests a competitive relationship between cross-linking reactions and tar evolution during pyrolysis. Following radical formation, two principal reaction pathways are possible: first, cleavage of radicals to form small volatile molecules that contribute to tar and gas products; second, recombination via cross-linking reactions, which generate new covalent bonds and progressively reinforce the solid carbon matrix.

### 3.3. The Distribution of Gas Products During Pyrolysis

**Figure 4:**
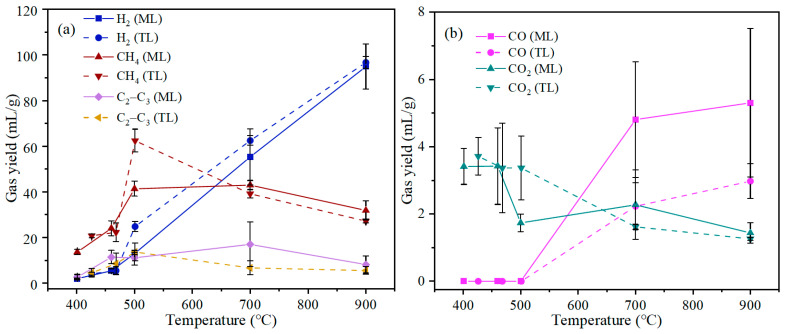
Gas product yields of two coking coals under different temperatures during pyrolysis: (**a**) H_2_, CH_4_, and C_2_–C_3_ hydrocarbons; (**b**) CO, CO_2_.

The gas product yields of ML and TL coal samples were approximately 3% at initial temperature and 12.19–14.01% at 900 °C from Figure 3. GC equipment was used to analyze the gas composition, including H_2_, CH_4_, CO, CO_2_, and C_2_–C_3_ hydrocarbons (C_2_H_6_, C_2_H_4_, C_3_H_8_), and the results are shown in Table 2 and Figure 4.

During coal pyrolysis, cross-linking reactions constitute a critical link between the evolution of gaseous products and the structural transformation of the solid phase. These reactions involve the recombination and condensation of free radicals, leading to the formation of a more compact and highly aromatic carbon network. The release of light gases offers direct evidence for the initiation and intensity of cross-linking processes. Figure 4 illustrates the influence of pyrolysis temperature on the composition of gaseous products from ML and TL coals. The gases evolved during pyrolysis may originate either from the direct decomposition of macromolecular structures, cross-linking reactions, or from secondary reactions of volatile intermediates [6,25]. In this study, the evolution of H_2_, CH_4_, CO, CO_2_, and C_2_–C_3_ hydrocarbons was examined in detail. When the temperature exceeded 700 °C, the yields of H_2_ and CO showed a marked increase. In particular, the release of H_2_ rose significantly with increasing temperature. It has been reported that H_2_ formation during pyrolysis results mainly from two pathways: radical polycondensation and dehydrogenation [12,25]. At 700 °C, the H_2_ yields for ML and TL coals were 55.3 and 62.49 mL/g, respectively, indicating enhanced condensation of aromatic structures accompanied by hydrogen release, which promotes further aromatization. At 900 °C, the H_2_ yield increased dramatically to 94.93 and 96.72 mL/g of these two coals, becoming the predominant gaseous product. This notable rise is closely associated with deep dehydrogenation of aromatic rings and secondary cracking reactions under high-temperature conditions. The sharp increase in H_2_ yield above 700 °C, which was consistent with increased aromatization/dehydrogenation that accompanies cross-linking, during which extensive dehydrogenative condensation of aromatic radicals takes place, resulted in the formation of new C–C bonds and the release of H_2_. The higher H_2_ yield observed for TL coal at elevated temperatures suggests a greater extent of cross-linking and aromatic condensation, ultimately leading to a more ordered and compact char structure. In terms of gas composition, H_2_ and CH_4_ represented major components, especially at higher temperatures. The generally higher yields of H_2_ from TL coal compared to ML coal may reflect higher cross-linking, as shown in the literature [6]. While the CH_4_ yield of TL coal was observed to increase particularly at 500 °C compared to ML coal, the result indicated that TL coal undergoes cross-linking at 500 °C. Although ML coal possesses a higher volatile matter content (Table 1), its lower H_2_ and CH_4_ production relative to TL coal could be attributed to its greater fluidity, which maintained a gas–liquid–solid phase during pyrolysis, potentially suppressing certain condensation and cross-linking pathways. In conclusion, these findings provide valuable insights into the mechanisms of coal pyrolysis and offer a basis for the targeted regulation of gas product distributions.

CH_4_ and C_2_–C_3_ hydrocarbons are primarily generated from the cleavage of aliphatic side chains, bridge bonds, and aromatic side chains, as well as from the methyl groups in alkanes [26]. During the pyrolysis of TL coal, the yields of CH_4_ and C_2_–C_3_ hydrocarbons initially increased due to cross-linking reactions [6], followed by a decrease attributed to secondary cracking at elevated temperatures. Specifically, C_2_–C_3_ hydrocarbons from TL coal increased up to 700 °C and then showed a slight decline at 900 °C, indicating their further decomposition into smaller molecules such as H_2_ and CH_4_ under high-temperature conditions. With increasing temperature, cross-linking reactions gradually become more competitive with chain scission by consuming reactive radicals. The relatively higher yields of CH_4_ and C_2_–C_3_ hydrocarbons from ML coal at high temperatures suggest that radical consumption via side-chain cracking remained significant. This implies that cross-linking reactions were less dominant and occurred at a relatively later stage in ML compared to TL. It was possible that during the formation of the plastic layer, radicals were preferentially involved in reactions that contributed to the maintenance of fluidity.

The formation of CO was closely linked to the involvement of oxygen-containing functional groups in cross-linking reactions. CO was detected at temperatures above 700 °C and increased with rising temperature, indicating the decomposition of oxygen-containing functional groups (such as ether and carbonyl groups) upon reaching elevated temperatures. At high temperatures, the rearrangement or decomposition of carbonyl and ether structures can release CO. The higher CO yield observed for ML in the high-temperature range suggests a greater participation of oxygen functional groups in cross-linking processes, thereby promoting the development of a more complex and interconnected carbon structure.

**Figure 5:**
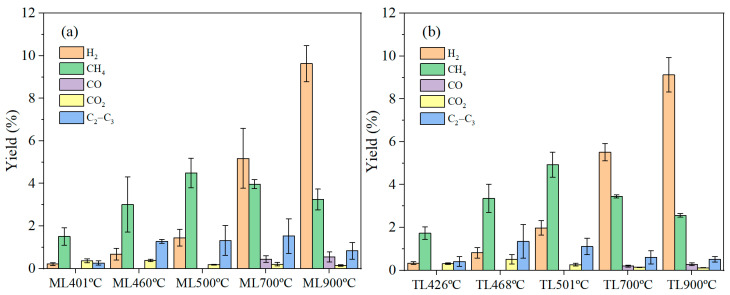
Gas products changes in coking coals under different temperatures during pyrolysis: (**a**) ML; (**b**) TL.

The pyrolysis products for each temperature were normalized, the gas product concentration changes in two coking coals for each temperature during pyrolysis are obtained in Figure S2, and the yield of gases is shown in Figure 5. During the pyrolysis process, the varieties of gaseous products generated from the two coking coals and their evolution trends with temperature are largely comparable. At initial pyrolysis stages, CH_4_ and H_2_ are the main gaseous products, while the yields of CO, CO_2_, and C_2_–C_3_ are very low. This indicates that the dominant reactions in this stage involve the cleavage of aliphatic side chains and weak bonds in coal, generating small-molecule hydrocarbon gases. As shown in Figure S2, at temperatures below 500 °C, CH_4_ accounts for over 60% of the gaseous products. Above 700 °C, H_2_ and CH_4_ continue to be the major gaseous species. Notably, TL coal exhibits higher H_2_ yields at lower temperatures, indicating that ML coal may contain a greater amount of transferable hydrogen, which can play a significant role during the plastic-layer stage [12,27]. As temperature increases, the concentration of H_2_ increases markedly, especially above 700 °C, reflecting the growing contribution of dehydrogenative condensation and aromatic cross-linking reactions. The yield of CH_4_ initially increases with temperature and then declines. Compared with TL, ML coal maintains relatively lower CH_4_ and C_2_–C_3_ yields below 700 °C, but higher yields at elevated temperatures. This result implied that a considerable portion of reactive radicals in ML coal continues to be consumed by side-chain cleavage pathways and released the light gases above 700 °C. Such behavior means that cross-linking reactions occurred and formatted more aromatic structure. ML coal was associated with higher fluidity and vitrinite content, which facilitate reactions dominated by cracking and delay the predominance of cross-linking [12,28,29]. With increasing temperature, the CO_2_ yield from both ML coal and TL coal displayed a declining trend. CO was detected only at temperatures above 700 °C and remained at a relatively low concentration in the pyrolysis gas.

### 3.4. The Distribution of Tar Products During Pyrolysis

#### 3.4.1. The Distribution of Tar Products with Different Temperatures

**Figure 6:**
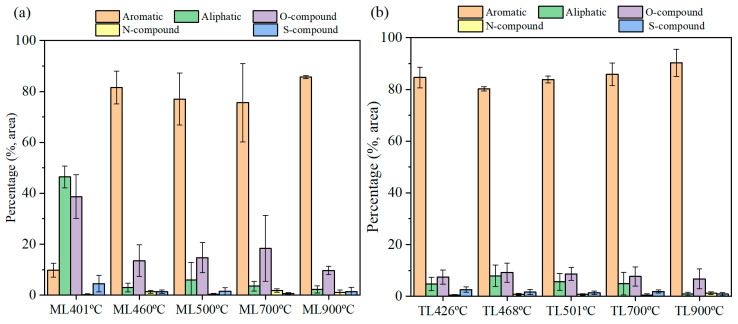
Tar products of different coking coals under different temperatures during pyrolysis: (**a**) ML; (**b**) TL.

**Figure 7:**
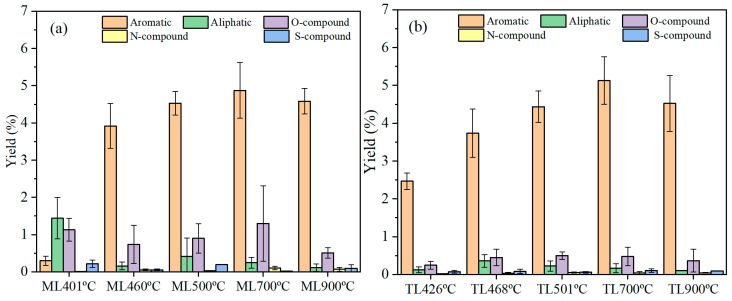
Tar products yield of different coking coals at different temperatures during pyrolysis: (**a**) ML; (**b**) TL.

Each coal sample was pyrolyzed at 400–500 °C, 700 °C, and 900 °C, respectively, and obtained tar components which were analyzed by GC–MS. Tar is mainly composed of aromatic hydrocarbons, aliphatic hydrocarbons, oxygen-containing compounds, and heterocyclic compounds containing N, S, and other heteroatoms. Figure 6a,b shows the tar product distribution of ML and TL coals with varying temperatures. Figure 7a,b shows the tar product yields of ML and TL coals at different temperatures.

Figure 6a shows the composition of tar derived from ML at different pyrolysis temperatures. At the initial pyrolysis temperature of 401 °C, oxygen-containing compounds (38.7%) and aliphatic hydrocarbons (46.4%) constitute the predominant components of ML coal tar, while aromatic hydrocarbons account for only approximately 10%. With further elevation of temperature, the proportion of aromatic hydrocarbons in ML tar shows a consistent increase. With the temperature increasing, the yield of aromatic hydrocarbons increased, whereas the content of aliphatic hydrocarbons declines significantly to below 6%. The content of oxygen-containing compounds, in contrast, exhibits a fluctuating pattern. Upon increasing the pyrolysis temperature to 460 °C, the concentrations of oxygen-containing compounds (13.53%) and aliphatic hydrocarbons (3%) in ML tar decrease sharply, while the aromatic hydrocarbon content rises markedly to 81.6%. At 900 °C, the aromatic hydrocarbon content attains its peak value of approximately 86%.

As shown in Figure 6b, in contrast to ML coal, the tar derived from TL coal contains more than 80% aromatic hydrocarbons across the entire range of pyrolysis temperatures. Oxygen-containing compounds constitute the second most abundant fraction and remain relatively stable at about 10%, while the content of aliphatic hydrocarbons stays below 8%. When the pyrolysis temperature increases to 468 °C, the contents of oxygen-containing compounds and aliphatic hydrocarbons in TL tar increase slightly, accompanied by a marginal decrease in aromatic hydrocarbons. After the resolidification temperature, the aliphatic hydrocarbon content decreases gradually, whereas the contents of the other components show no significant variation.

Significant differences are observed between the tar products derived from the two coals, which provide important insights into their respective pyrolysis mechanisms. At the initial pyrolysis temperature, the tar from ML coal is predominantly composed of oxygen-containing compounds and aliphatic hydrocarbons. This composition may be explained by the substantial release of aliphatic hydrocarbons and alkanes at relatively low temperatures during the initial softening stage, likely associated with the higher fluidity of ML coal. When pyrolysis is conducted at the temperature of maximum fluidity (460 °C), the aromatic hydrocarbon content in ML tar reaches 81.6%, followed by oxygen-containing compounds at 13.5%. This result indicates that during the plastic layer formation stage, there is no excessive release of aliphatic and oxygen-containing species; rather, a considerable portion of these components likely remains within the plastic layer in a gas–liquid–solid coexistent state without being evolved.

At a resolidification temperature of 500 °C, the aromatic hydrocarbon content in ML tar attains 77%, while oxygen-containing compounds constitute 14.7%. During the semi-coke solidification stage, few significant releases of aliphatic hydrocarbons are detected, implying that aliphatic structures are incorporated into the semi-coke solidification process and promote the development of a more stable carbon architecture. Although oxygen-containing compounds do not evolve during the plastic layer formation stage, they are released following resolidification. This indicates that while such compounds contribute to the softening and melting phases, they do not partake in the solidification process that ultimately leads to semi-coke formation.

At a pyrolysis temperature of 700 °C, the content of oxygen-containing compounds in ML tar increases, implying that part of the tar undergoes cross-linking reactions with the solid semi-coke after resolidification, while the remaining oxygen-containing compounds are released in the form of tar and gaseous products. This observation is consistent with the relatively higher CO content in the gaseous products of ML coal compared with TL coal. At 900 °C, the aromatic hydrocarbon content in ML tar increases to approximately 85.6%, whereas the oxygen-containing compounds decrease to 9.7%. In contrast, the contents of various components in TL tar decrease slightly at 900 °C, which may be attributed to secondary cracking reactions leading to a reduction in tar yield. As shown in Figure 7b, the proportion of aromatic hydrocarbons in TL tar remains consistently high at different pyrolysis temperatures.

Figure 7a,b show the yield of tar products per gram of coking coal during pyrolysis at different temperatures. The results reveal distinct variations in tar yield depending on the coal composition and pyrolysis temperature. As the temperature increased, the tar yield exhibited a progressive rise, accompanied by a gradual increase in the content of aromatic components within the tar. At 700 °C, the yield of aromatic components from both coals reached a peak, followed by a decline at 900 °C. This decrease was attributed to the decomposition of tar and char into gaseous products at elevated temperatures. Additionally, for the lower-rank coal ML, the yield of oxygen-containing compounds was higher than that of TL.

#### 3.4.2. The Aliphatic and Aromatic Compounds of Tar Products

**Figure 8:**
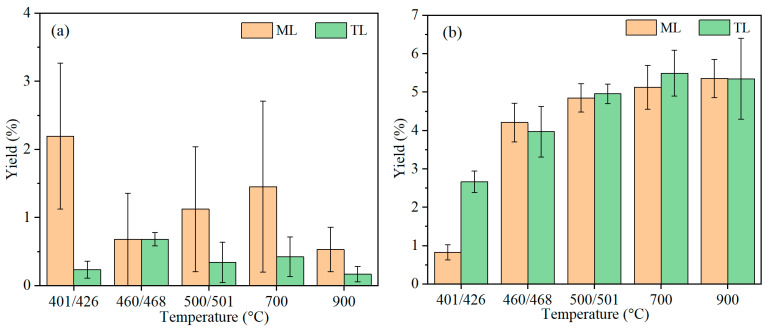
Tar product changes in coking coals with temperatures during pyrolysis: (**a**) aliphatic; (**b**) aromatic.

The aliphatic and oxygen-containing structures in tar products play a critical role in the mechanism of coal pyrolysis. Oxygen-containing species (such as alcohols, esters, acids, ketones, phenols, ethers, and aldehydes) encompass both aliphatic and aromatic configurations. To systematically investigate whether the overall distribution of aromatic and aliphatic structures influences the pyrolysis process, these components were categorized into aromatic and aliphatic compounds. Figure 8a,b shows the resulting variations in the yields of aromatic compounds and aliphatic compounds released respectively in the tar from the two coking coals at different temperatures. The aliphatic compounds include alkanes, alkenes, and aliphatic oxygen-containing compounds. The aromatic compounds comprise monocyclic aromatics (e.g., benzene derivatives), polycyclic aromatic hydrocarbons (PAHs) such as naphthalene, phenanthrene, and anthracene, as well as oxygenated aromatic species.

As shown in Figure 8a, ML coal evolved a significant proportion of aliphatic structures at the initial softening temperature. However, upon reaching the temperature of maximum fluidity, the aliphatic content in ML coal decreased markedly and remained the lowest, while the release of aliphatic structures from TL coal increased substantially and showed the highest content. From Figure 8a, the aliphatic structures of ML coal were released vibratingly even at the same temperature. This difference indicates that in ML coal, aliphatic structures are predominantly involved in solid-phase chemical reactions and cross-linking reactions during plastic mass formation at the temperature of maximum fluidity, rather than being released as volatiles. In contrast, TL coal with lower aliphatic compounds releases aliphatic components more largely at the temperature of maximum fluidity, and then the release decreases with temperature through conventional volatile evolution during pyrolysis. At resolidification temperatures of 500 °C and 700 °C, the aliphatic tar yield of ML coal increases. This result suggested that a portion of the aliphatic structures fail to integrate into the semi-coke matrix following solidification and was subsequently released as tar products. At 900 °C, the decrease in aliphatic products was likely attributable to secondary decomposition. Via the repeated experimental results, the larger fluctuation in aliphatic structure in tar can be found. It means the aliphatic structure in coal for the mechanism of coking is very important.

Figure 8b further illustrated that the yield of aromatic compounds in tar increased significantly with rising temperature for two coals. At the initial softening temperature, ML coal produced only a small quantity of aromatic compounds, whereas TL coal released a greater amount than ML coal. Above the resolidification temperature of approximately 500 °C, the variation in aromatic compounds became less pronounced, as the decomposition process was essentially complete. This indicates that tar formation primarily occurs below the resolidification temperature. Beyond 700 °C, a slight increase in tar yield is observed, which can be attributed to the formation and rearrangement of aromatic structures during pyrolysis as the temperature continues to rise.

#### 3.4.3. The Distribution of Aromatic Compounds in Tar Products

Aromatic hydrocarbon compounds, including benzene, biphenyl, indene, naphthalene (Nap), phenanthrene (PhA), anthracene (AnT), fluorene (Flu), acenaphthene (AcP), pyrene (Pyr), fluoranthene (FluA), chrysene (Chr), benzo[a]pyrene (benzo[a]P., BaP), benzo[a]anthracene (Benzo[a]A., BaA) and benzo[b]fluoranthene (benzo[b]F., BbF), among others, were investigated in this study. The aromatic hydrocarbons distribution of coking coals (ML and TL coal) with different temperatures during pyrolysis is shown in Figure S3. As observed, the concentration of each aromatic hydrocarbon varied with pyrolysis temperature. Naphthalene was identified as the most abundant aromatic hydrocarbon at all temperatures, followed by phenanthrene. This indicates that two-ring and three-ring aromatic hydrocarbons constitute the dominant components under the experimental conditions.

**Figure 9:**
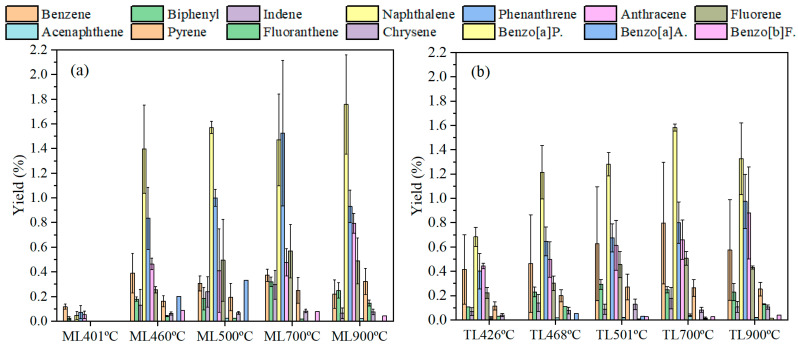
Variations in aromatic hydrocarbon yields with temperature during pyrolysis of ML and TL coals: (**a**) ML; (**b**) TL.

The variations in aromatic hydrocarbon yields with temperature during the pyrolysis of ML and TL coals is shown in Figure 9. It can be seen that the release profiles of the 12 detected PAHs differ between the two coals, with naphthalene, phenanthrene, and anthracene representing the predominant compounds. A notable distinction was the significantly lower yield of aromatic compounds from ML coal at the onset of softening. This result may be due to the lower tar generation at its initial decomposition temperature, or higher aliphatic chain possibly related to ML coal’s relatively higher Gieseler fluidity and vitrinite content. Although vitrinite is known to produce substantial aromatic structures [30], these components may not be released immediately from the plastic layer during pyrolysis and thus were not captured in the tar phase, a finding consistent with the established influence of vitrinite on coking behavior. In contrast, TL coal exhibited detectable release of certain aromatic components even at the initial temperature. At the maximum fluidity temperatures (460 and 468 °C), ML coal and TL coal primarily generated greater amounts of naphthalene and phenanthrene derivatives mainly. This result suggests that two-ring and three-ring aromatic compounds play a significant role in influencing the fluidity of coking coal [14], whereas monocyclic aromatics have only a minor effect. Following resolidification, the yields of naphthalene, phenanthrene, and anthracene from ML coal were slightly higher than those from TL coal, suggesting that TL coal undergoes more extensive cross-linking reactions during the post-solidification stage.

### 3.5. Changes in Carbon Structures During Coking Pyrolysis

#### 3.5.1. XPS Analysis of Carbon Structures

**Figure 10:**
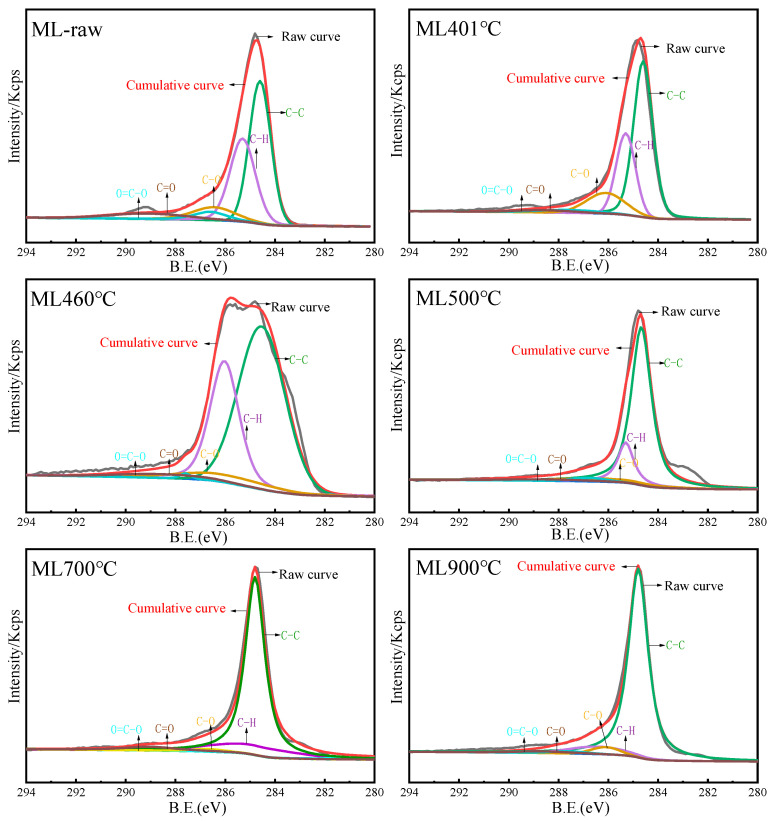
XPS spectrum and the curve fitted of ML coal and its semi-coke under different temperatures.

The C 1s spectra were deconvoluted into five components based on the assignments summarized in Table S1. Representative XPS spectra of the ML coal and its semi-cokes obtained at temperatures ranging from 400 to 900 °C are shown in Figure 10, while the corresponding spectra and peak-fitting results for the TL coal series are provided in Figure S4. Notable changes were observed in the spectral line shapes depending on the treatment temperature. As shown in Figure 10 and Figure S4, the C 1s spectrum of the ML sample treated at 460 °C exhibited a noticeable broadening, which can be attributed to the emergence of aliphatic C–H species. In contrast, the TL sample treated at the same temperature displayed a distinct shoulder peak, indicating a similar trend in spectral evolution but with variations in the relative contributions of different carbon functional groups. These results suggest that at the temperature corresponding to maximum fluidity, the ML coal developed abundant C–H aliphatic structures, whereas the TL coal was characterized by a higher proportion of C–O bonds at this stage.

**Figure 11:**
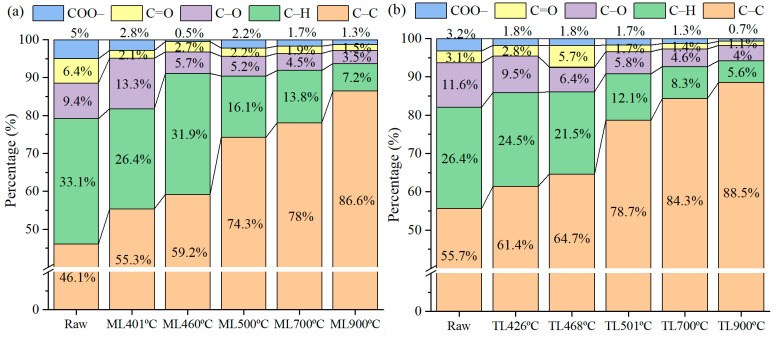
Aromatic hydrocarbon changes in coking coals under different temperatures during pyrolysis: (**a**) ML; (**b**) TL.

The assignment and relative proportions of the C1s peaks for the ML and TL samples are summarized in Table S2. Figure 11a,b illustrates the contents of C–C, C–H, C–O, C=O, and O=C–O functional groups of ML and TL coals. It can be observed that the proportion of aromatic C–C bonds increased with rising temperature [7]. TL coal exhibited a higher C–C content compared to ML coal, which is consistent with its higher rank and greater carbon content as determined by elemental analysis. Additionally, elemental analysis indicated that ML coal contains higher concentrations of oxygen and hydrogen, corresponding to its more pronounced C–H, C–O, C=O, and O=C–O spectral bands relative to TL coal. Overall, a larger release of CO and CO_2_ was detected from ML coal than from TL coal during pyrolysis. The C–O was improved at the maximum Gieseler fluidity temperature, and perhaps carbon dioxide can be reduced to carbon monoxide through gas–solid reactions. Oxygen atoms played a significant role in influencing the cross-linking of aromatic structures. It was suggested that ML coal contains more oxygen-containing bridge bonds (e.g., –O–, –CH_2_–O–, etc.), alongside an increase in stable free radical concentration [16]. Beyond 500 °C, the notable rise in aromatic C–C content indicated that solidification promoted the cross-linking of aromatic structures.

ML coal exhibited a higher content of C–H structures compared to TL coal. As shown in Figure 11, the relative C–H content of ML coal was increased at 460 °C, corresponding to its maximum fluidity temperature. Conversely, Figure 6 and Figure 7 revealed a decline in aliphatic hydrocarbons in the tar. This apparent discrepancy, namely an increase in C–H content of the char and reduced aliphatic hydrocarbon release of the tar, can be attributed to cross-linking reactions occurring in ML coal around the maximum fluidity temperature, which suppress aliphatic hydrocarbon evolution. These results further indicated that cross-linking in ML coal initiates at relatively low temperatures. Below 500 °C, cleavage of bridge bonds in longer aliphatic side chains, along with decomposition of carboxyl groups and ether bonds, takes place. The yields of H_2_, CH_4_, and C_2_–C_3_ components from ML coal pyrolysis are lower than those from TL coal, while the CO and CO_2_ yields from the two coals are comparable from Table 2. Although ML coal with low-rank typically generates larger amounts of aliphatic volatile products, the relatively lower release of aliphatic volatiles (H_2_, CH_4_, and C_2_–C_3_) observed in ML coal may be associated with cross-linking reactions occurring during the maximum fluidity stage. Beyond the resolidification temperature, the relative content of aromatic C–C bonds gradually increases at the expense of C–H structures, indicating progressive condensation and cross-linking that enhance the structural ordering of carbon microcrystals. This trend is consistent with the result of related literature [7,12]. Meanwhile, oxygen-containing functional groups (C–O, C=O, O–C=O) decrease significantly, reflecting decarboxylation and deoxygenation reactions accompanied by the release of volatiles such as CO, CO_2_, and oxygen-containing compounds. ML coal exhibits higher initial contents of oxygen-containing functionalities and C–H groups than TL coal, consistent with its lower rank and elevated H/C and O/C ratios. At the maximum fluidity temperature (460 °C), an increase in C–H content is observed for ML coal, suggesting enhanced cross-linking reactions [31]. Above 500 °C, the transformation of C–H and C–O bonds into aromatic C–C structures for TL coal became dominant, marking the onset of resolidification and the development of a more condensed aromatic network [7].

#### 3.5.2. Correlation Between XPS-Derived Carbon Structures and H_2_ Evolution

**Figure 12:**
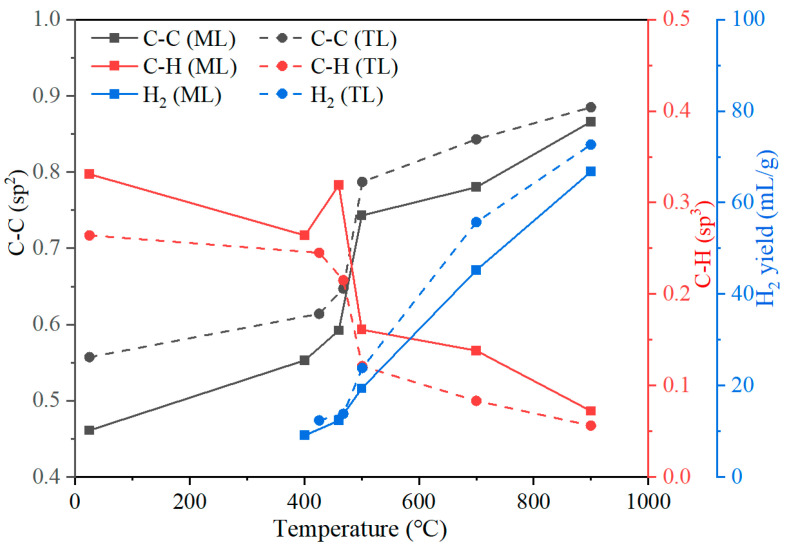
Correlation between (C–C)/(C–H) and H_2_ evolution during pyrolysis.

As shown in Figure 11 and Table S2, an increase in pyrolysis temperature to 900 °C, led to a rise in the proportion of C–C(sp^2^) bonds in the char from 46–56% to 87–89%, accompanied by a marked decrease in C–H components from 26–33% to 6–7%. Figure 12 shows that the evolution of carbon chemical states (C–C/C–H) correlates with H_2_ evolation during pyrolysis.

As shown in Figure 12, as the temperature increases, the proportion of C–C bonds gradually increased. Notably, above the resolidification temperature, the increase in C–C bonds became significantly more pronounced. This suggests that the char undergoes progressive enrichment in aromatic ring structures after resolidification, leading to a more ordered and condensed carbon framework. Concurrently, the proportion of C–H bonds declines, indicating the progressive removal of aliphatic side chains or hydrogen atoms from the solid surface. The decreasing trend in C–H bonds aligns with the increasing trend in C–C bonds, further confirming that hydrogen was gradually stripped from the solid char structure at elevated temperatures, thereby facilitating the formation of larger aromatic structures. The released hydrogen radicals were subsequently recombined and released as H_2_ [32]. The released H_2_ increases substantially with temperature, particularly above 500 °C, with the maximum release rate observed at around 700 °C.

It is also noteworthy that, for ML coal, the C–H content showed a slight increase near the temperature of maximum fluidity. This suggests that aliphatic C–H bonds do not immediately volatilize during the formation of the plastic layer, but instead remain within the system in a liquid–solid coexistence state, thereby contributing to the observed increase. This observation aligns with the results shown in Figure 7, where the release of aliphatic hydrocarbons in the tar from ML coal at 460 °C was relatively low. Furthermore, this phenomenon may also reflect the occurrence of cross-linking reactions in ML coal during pyrolysis within the temperature range of 400 to 500 °C.

Following the resolidification stage, hydrogen evolution is primarily attributed to the dehydrogenation of aliphatic groups and the condensation of aromatic structures. During this phase, the progressive removal of hydrogen atoms from the carbon matrix facilitates the transformation of C–H bonds into C–C bonds, thereby promoting the growth of larger aromatic clusters and enhancing the structural ordering of carbon at elevated temperatures [7]. It was indicative of cross-linking mechanisms during coal pyrolysis.

**Figure 13:**
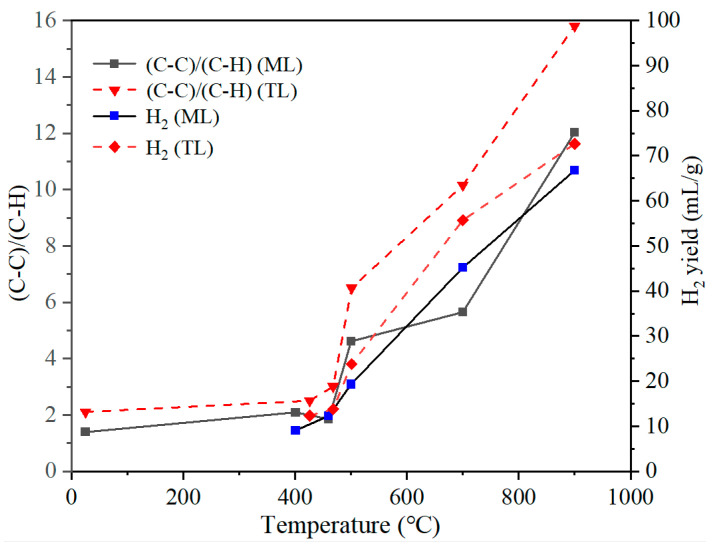
Change in the (C–C)/(C–H) ratio derived from XPS and its correlation with H_2_ yield with temperature.

Variation in the (C–C)/(C–H) ratio derived from XPS and its relationship with H_2_ evolution during pyrolysis is shown in Figure 13. It can be seen that the (C–C)/(C–H) ratios of the two coals remain relatively unchanged at the initial softening temperature. At the temperature corresponding to maximum fluidity, the ratios exhibit a slight increase. Following resolidification, a marked increase in the (C–C)/(C–H) ratio was observed. With further increase in pyrolysis temperature, the (C–C)/(C–H) ratio continues to increase significantly; however, the (C–C)/(C–H) ratio for TL coal is consistently higher than those for ML coal throughout the process. This discrepancy may be attributed to differences in the structural evolution behaviors of the two coals during pyrolysis.

A comparative analysis of the variation in the (C–C)/(C–H) ratio with the H_2_ evolution trend for each coal reveals a strong correlation between changes in the (C–C)/(C–H) ratio and hydrogen release during pyrolysis. As shown in Figure S5, the (C–C)/(C–H) ratio exhibits a clear positive correlation with H_2_ generation, indicating that the evolution of this structural parameter is closely linked to the release of hydrogen. Notably, both the aromatic condensation degree and the hydrogen release behavior in ML coal lag behind those observed in TL coal, suggesting distinct reaction pathways during the coking process. Specifically, TL coal undergoes a relatively continuous and smoother structural transformation, whereas ML coal is likely subject to more pronounced cross-linking reactions during pyrolysis, thereby delaying the evolution of hydrogen.

#### 3.5.3. SEM Analysis of Carbon Structures

**Figure 14:**
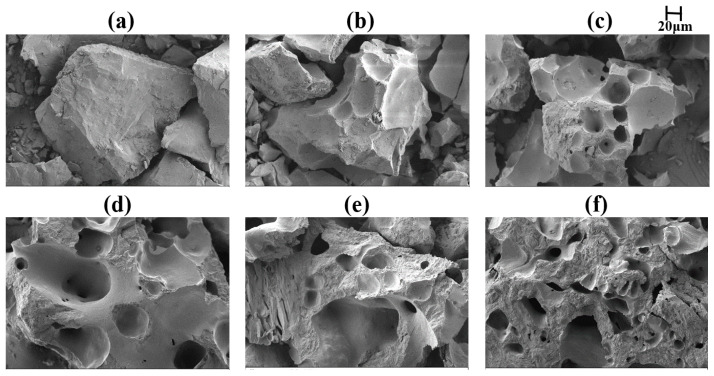
SEM morphology of ML sample from different temperatures during pyrolysis: (**a**) raw coal of ML coal; (**b**) 401 °C; (**c**) 460 °C; (**d**) 500 °C; (**e**) 700 °C; (**f**) 900 °C.

SEM morphology of ML coal and the semi-coke at different temperatures during coking is shown in Figure 14. Figure 14a shows that the raw ML coal sample consists of individual coal particles. In Figure 14b, captured at the onset of thermal decomposition, the sample contains a mixture of unaltered, deformed, and softened coal particles. Figure 14c corresponds to the temperature of maximum fluidity, where a well-developed porous structure with numerous quasi-spherical bubbles is evident. At this stage, the coal undergoes intense reactions accompanied by the evolution of gas, liquid, and solid phases. The material becomes fragmented and transitions into a plastic mass, effectively forming a fusion zone as the particles melt. Bubble formation results from the expansion of the plastic layer, which generates internal swelling pressure [10]. As shown in Figure 14d, the sample morphology after extensive decomposition and subsequent solidification is characterized by the development of larger layered structures and macropores produced by the release of volatile matter. Figure 14e indicates that the structure at 700 °C, where the semi-coke exhibits a denser morphology with reduced pore size. Figure 14f illustrates the sample at 900 °C, approaching fully formed coke, which displays a more compact microstructure. As shown above, these observations delineate the structural progression from raw coal through the plastic state to semi-coke during coking. The development of the plastic state is critical for coal caking and is fundamentally linked to transformative changes in the chemical structure.

## 4. Conclusions

The product yield distribution, gas evolution characteristics, and tar composition analysis of two coking coals (ML and TL) during pyrolysis was systematically investigated. The behavior and cross-linking structural evolution were studied using GC, GC-MS, XPS, and SEM analysis.

Temperature and the characteristics of coals played an important role in regulating the competition between chain cleavage and cross-linking reactions. During the plastic layer formation stage (below 500 °C), a reduction in aliphatic groups in ML tar was observed, while a transient increase in C–H bonds of char near the temperature of maximum fluidity occurred. ML coal, characterized by higher fluidity and greater volatile content, exhibited pronounced retention of aliphatic structures within the plastic layer. TL coal displayed more conventional volatile release behavior. It indicates that cross-linking reactions play a predominant role in the transformation of ML coal at this stage.

After resolidification temperatures, TL coal yielded more H_2_ and developed more stable aromatic structures. The substantial increase in H_2_ evolution, the growth of aromatic C–C structures and C-H decreased evidenced by XPS. The (C–C)/(C–H) ratio exhibits a positive correlation with H_2_ release and serves as a key indicator for characterizing the degree of aromatization cross-linking during coal pyrolysis. The progressive densification observed by SEM collectively confirm the intensification of cross-linking reactions.

Given that cross-linking during coking pyrolysis renders the process not a single-step reaction but a temperature-dependent competitive mechanism, a comprehensive understanding of coal pyrolysis requires the integration of more advanced analytical characterization techniques, such as in situ FT-IR, for real-time analysis.

## Figures and Tables

**Table 1:** Proximate analysis, ultimate analysis, and properties of coal samples.

Coal Samples		ML	TL
Proximate analysis (wt%, ad)	M	0.89	0.51
	A	9.19	10.23
	V	22.67	19.8
	FC	67.25	69.46
Ultimate analysis (wt%, daf)	C	86.79	88.4
	H	5.61	5.25
	N	2.13	1.75
	S	0.91	1.29
	O *	4.56	3.31
R_o max_		1.338	1.465
Gieseler Fluidity	T_i_ (°C)	401	426
	T_m_ (°C)	460	468
	T_r_ (°C)	500	501
	ΔT (°C)	99	75
	α_max_ (dd/min)	2500	500
Maceral analysis	Vitrinite (%)	60.2	48
	Inertinite (%)	36.4	48.9

ad—air dried basis; daf—dry ash free. * Differential calculation.

**Table 2:** Gas product yields of two coking coals under different temperatures during pyrolysis.

Samples	Temperature	H_2_	CH_4_	CO	CO_2_	C_2_–C_3_
		mL/g				
ML	401 °C	1.96 ± 0.27	13.61 ± 1.16	0.00 ± 0.00	3.41 ± 0.53	2.63 ± 1.19
	460 °C	5.44 ± 0.90	24.03 ± 3.29	0.00 ± 0.00	3.42 ± 1.14	11.40 ± 2.95
	500 °C	12.98 ± 0.41	41.36 ± 3.29	0.00 ± 0.00	1.73 ± 0.26	11.13 ± 3.18
	700 °C	55.30 ± 12.25	42.99 ± 2.00	4.81 ± 1.72	2.27 ± 1.03	17.03 ± 9.79
	900 °C	94.93 ± 9.93	31.87 ± 4.27	5.30 ± 2.21	1.43 ± 0.31	8.13 ± 3.69
TL	426 °C	4.09 ± 0.58	20.75 ± 0.81	0.00 ± 0.00	3.72 ± 0.56	4.55 ± 1.81
	468 °C	5.47 ± 1.69	22.32 ± 4.14	0.00 ± 0.00	3.37 ± 1.33	8.54 ± 4.70
	501 °C	24.81 ± 2.14	62.47 ± 5.01	0.00 ± 0.00	3.37 ± 0.95	13.66 ± 3.87
	700 °C	62.49 ± 2.08	39.16 ± 1.84	2.23 ± 0.70	1.62 ± 0.07	6.70 ± 3.08
	900 °C	96.72 ± 2.64	27.22 ± 0.80	2.97 ± 0.52	1.26 ± 0.04	5.55 ± 1.69

## Data Availability

The original contributions presented in this study are included in the article/Supplementary Materials. Further inquiries can be directed to the corresponding authors.

## Supplementary Materials

The following supporting information can be downloaded at https://www.mdpi.com/article/10.3390/ma19061096/s1, Table S1: Assignment and electron binding energy of peaks at C1s binding energy; Figure S1: TG-DTG results of the samples during pyrolysis: (a) TG; (b) DTG; Figure S2: Gas products changes in coking coals under different temperatures during pyrolysis: (a) ML; (b) TL; Figure S3: Aromatic Hydrocarbons distribution of coking coals with different temperatures during pyrolysis: (a) ML; (b) TL; Figure S4: XPS spectrum and the curve fitted of TL coal and its semi-coke under different temperatures; Table S2: Assignment and proportions of peaks for ML and TL sample at C1s binding energy; Figure S5: Change in the (C–C)/(C–H) ratio derived from XPS and its correlation with H_2_ yield.

## Abbreviations

The following abbreviations are used in this manuscript:

T_i_	Initial soft temperature of Gieseler Fluidity
T_m_	Maximum Gieseler fluidity temperature
T_r_	Resolidification temperature of Gieseler Fluidity
