# Supplementary material for: Insight into Pyrolysis Behavior and Cross-Linking Reactions Mechanism During Coking Coals Pyrolysis
Source: Materials (Basel). 2026 Mar 12;19(6):1096. doi: 10.3390/ma19061096 (PMC13027487; doi:10.3390/ma19061096)
Supplement: Supplementary file 1 [file materials-19-01096-s001.zip › materials-4174192-supplementary.pdf]

materials

MDPI

Article

Insight into Pyrolysis Behavior and Cross-linking Reactions Mechanism during Coking Coals Pyrolysis

Lu Tian [1], Jinxiao Dou [1,*], Xingxing Chen [1], and Jianglong Yu [1,2,*]

[1] Key Laboratory of Advanced Coal and Coking Technology of Liaoning Province, School of Chemical Engineering, University of Science and Technology Liaoning, Anshan 114051, China; 320163300148@ustl.edu.cn (L.T.); xingchenstar79@163.com (X.C.)
[2] Suzhou Industrial Park Monash Research Institute of Science and Technology, and Southeast University-Monash University Joint Graduate School, Suzhou 215123, China
* Correspondence: doujx123@163.com (J.D.); jianglongyu@163.com (J.Y.); Tel.: +86-(0)412-5929105 (J.Y.)

Supplementary Materials: The following supporting information can be downloaded at: https://www.mdpi.com/article/doi/s1, Table S1: Assignment and electron binding energy of peaks at C1s binding energy; Figure S1: TG-DTG results of the samples during pyrolysis: (a) TG; (b) DTG; Figure S2: Gas products changes of coking coals under different temperatures during pyrolysis: (a) ML; (b) TL; Figure S3: Aromatic Hydrocarbons distribution of coking coals with different temperatures during pyrolysis: (a) ML; (b) TL; Figure S4: XPS spectrum and the curve fitted of TL coal and its semi-coke under different temperatures; Table S2: Assignment and proportions of peaks for ML and TL sample at C1s binding energy; Figure S5. Change in the (C–C)/(C–H) ratio derived from XPS and its correlation with H_2 yield.

Table S1. Assignment and electron binding energy of peaks at **C1s** binding energy [7,16,21,22]

	Assignment		Electron binding energy (eV)
1	C-C	aromatic and graphitized carbon structure C–C	284.6
2	C-H	substituted alkanes C–H on the aromatic structure	285
3	C-O	the C–O in phenols and alcohols as well as ether C-O-C bonds	286.3
4	C=O	carbonyl C=O,	287.6
5	COO—	carboxyl COO– group	289.1
6	π-π*	π–π*	291

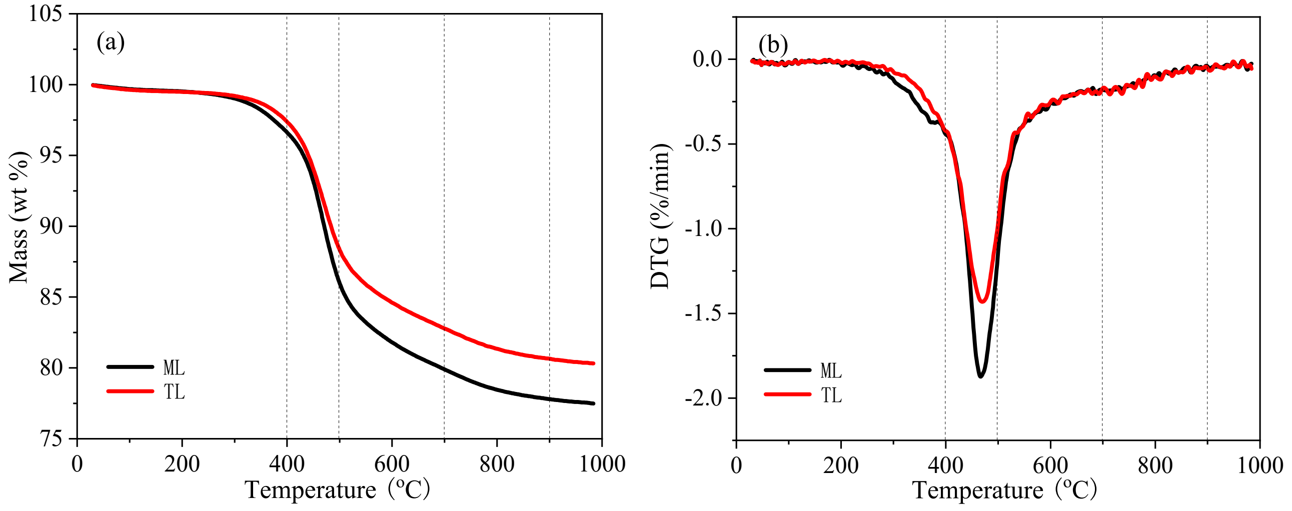

Figure S1. TG-DTG results of the samples during pyrolysis: (a) TG; (b) DTG

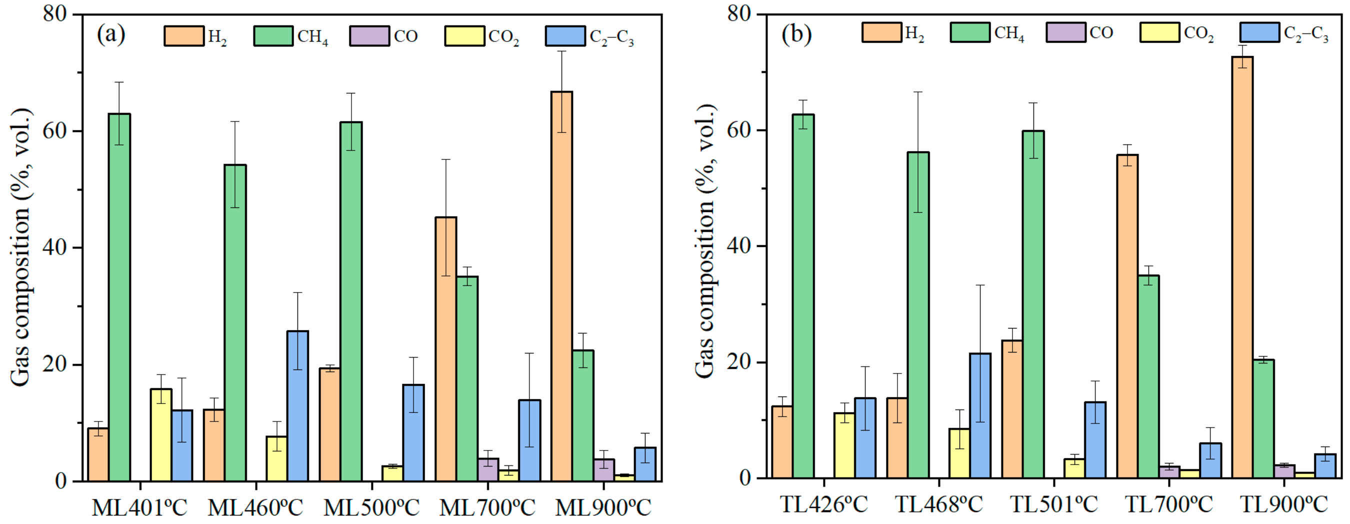

Figure S2. Gas products composition changes of coking coals under different temperatures during pyrolysis: (a) ML; (b) TL

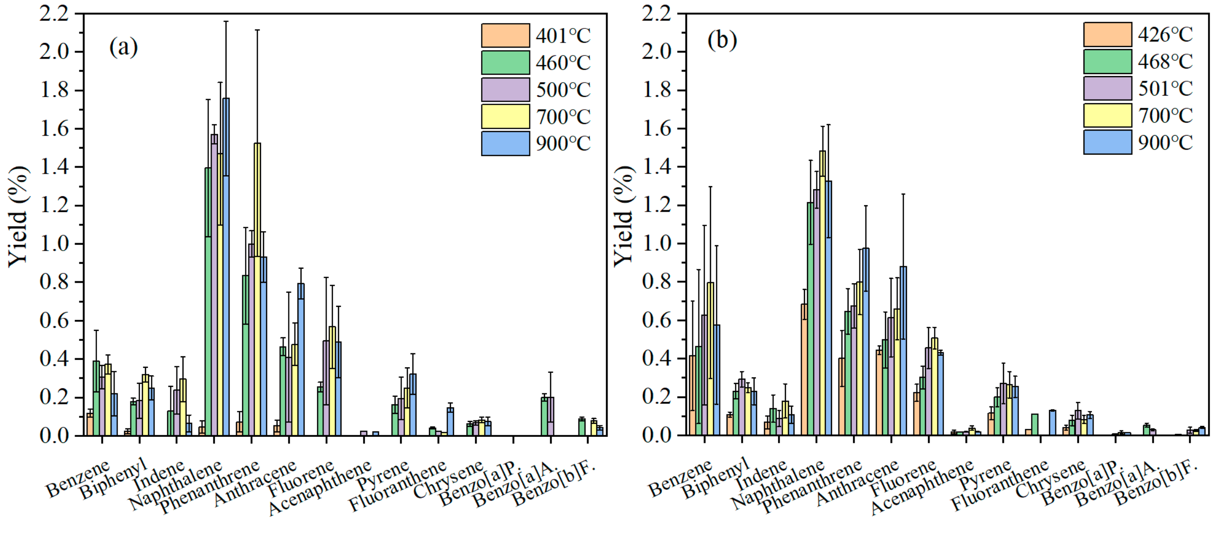

Figure S3. Aromatic Hydrocarbons distribution of coking coals with different temperatures during pyrolysis: (a) ML; (b) TL

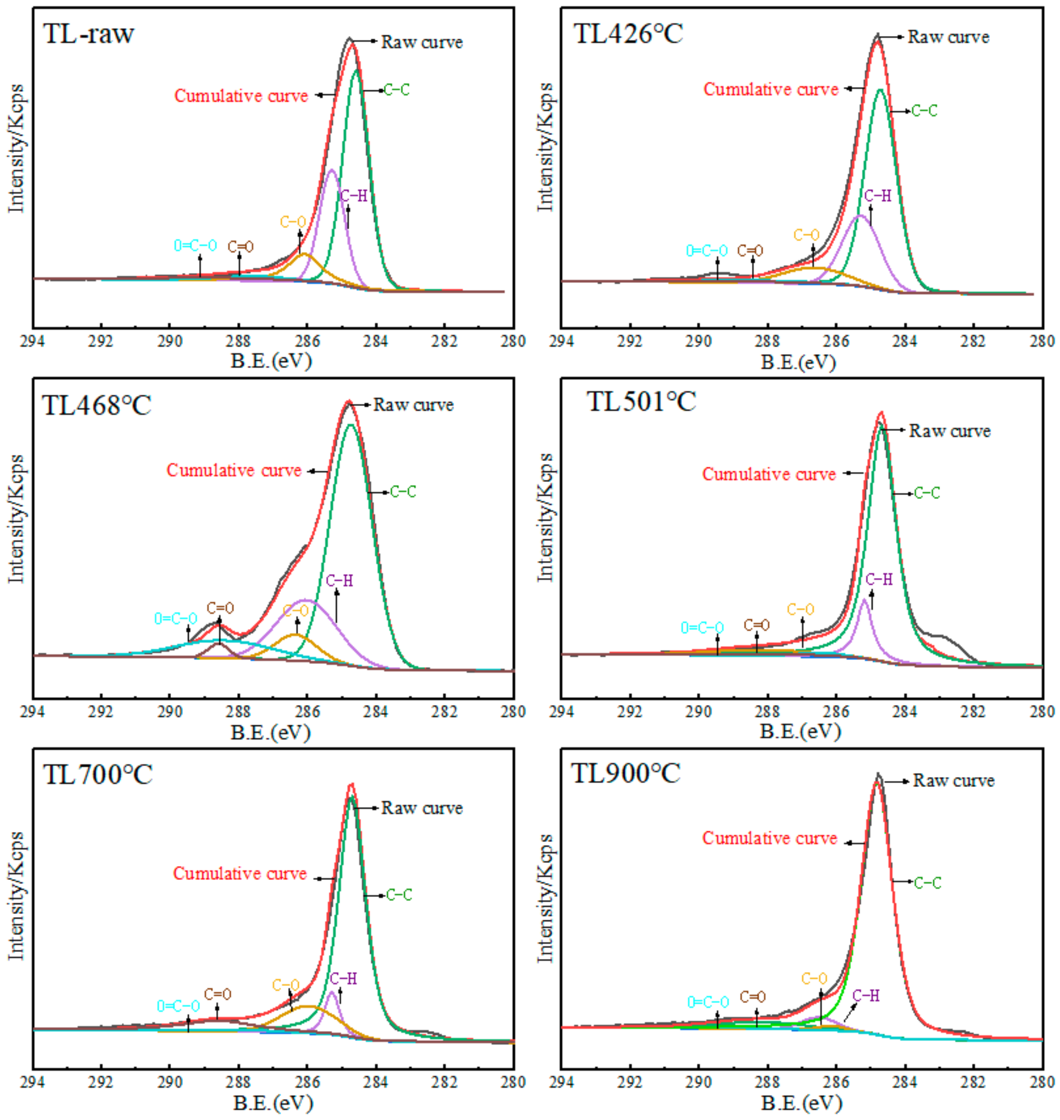

Figure S4. XPS spectra and the curve fitted of TL coal and its semi-coke under different temperatures

Table S2. Assignment and proportions of peaks for ML and TL sample at C1s binding energy

	C-C	C-H	C-O	C=O	O=C-O
ML-raw	0.461	0.331	0.094	0.064	0.050
ML401 °C	0.553	0.264	0.133	0.021	0.028
ML460 °C	0.592	0.319	0.057	0.027	0.005
ML500 °C	0.743	0.161	0.052	0.022	0.022
ML700 °C	0.780	0.138	0.045	0.019	0.017
ML900 °C	0.866	0.072	0.035	0.015	0.013
TL-raw	0.557	0.264	0.116	0.031	0.032
TL426 °C	0.614	0.245	0.095	0.028	0.018
TL468 °C	0.647	0.215	0.064	0.057	0.018
TL501 °C	0.787	0.121	0.058	0.017	0.017
TL700 °C	0.843	0.083	0.046	0.014	0.013
TL900 °C	0.885	0.056	0.040	0.011	0.007

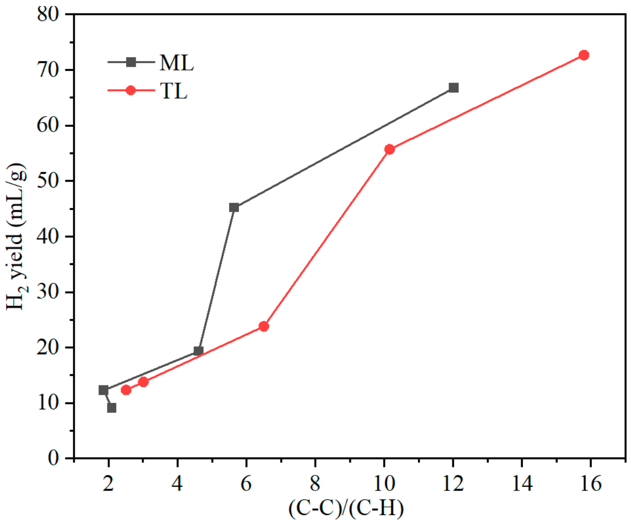

Figure S5. Change in the (C–C)/(C–H) ratio derived from XPS and its correlation with H_2 yield
